# Supplementary material for: Simultaneous Genetic Ablation of PD-1, LAG-3, and TIM-3 in CD8 T Cells Delays Tumor Growth and Improves Survival Outcome
Source: Int J Mol Sci. 2022 Mar 16;23(6):3207. doi: 10.3390/ijms23063207 (PMC8955581; doi:10.3390/ijms23063207)
Supplement: Supplementary file 1 [file ijms-23-03207-s001.zip › ijms-1574753-supplementary.pdf]

SUPPLEMENTARY MATERIAL

Simultaneous Genetic Ablation of PD-1, LAG-3, and TIM-3 in CD8 T Cells Delays Tumor Growth and Improves Survival Outcome

Author List and Affiliations:

Elisa Ciraolo[1]*, Stefanie Althoff [1]*, Josefine Ruß[1], Stanislav Rosnev[2,4], Monique Butze[1], Miriam Pühl[1], Marco Frentsch[3,4] Lars Bullinger[1,2,3,5], Il-Kang Na[1-5].

[1]Experimental and Clinical Research Center, Berlin, Germany.
[2]Department of Hematology, Oncology and Tumor Immunology, Charité-Universitätsmedizin Berlin, Corporate Member of Freie Universität Berlin, Humboldt-Universität zu Berlin, and Berlin Institute of Health, Berlin, Germany.
[3]Berlin Institute of Health (BIH), Berlin, Germany.
[4]Berlin Institute of Health Center for Regenerative Therapies, Charité-Universitätsmedizin Berlin, Berlin, Germany.
[5]German Cancer Consortium (DKTK), Berlin, Germany.

Content

Supplementary Figures 2

Supplementary Materials and Methods 5

Supplementary Figures

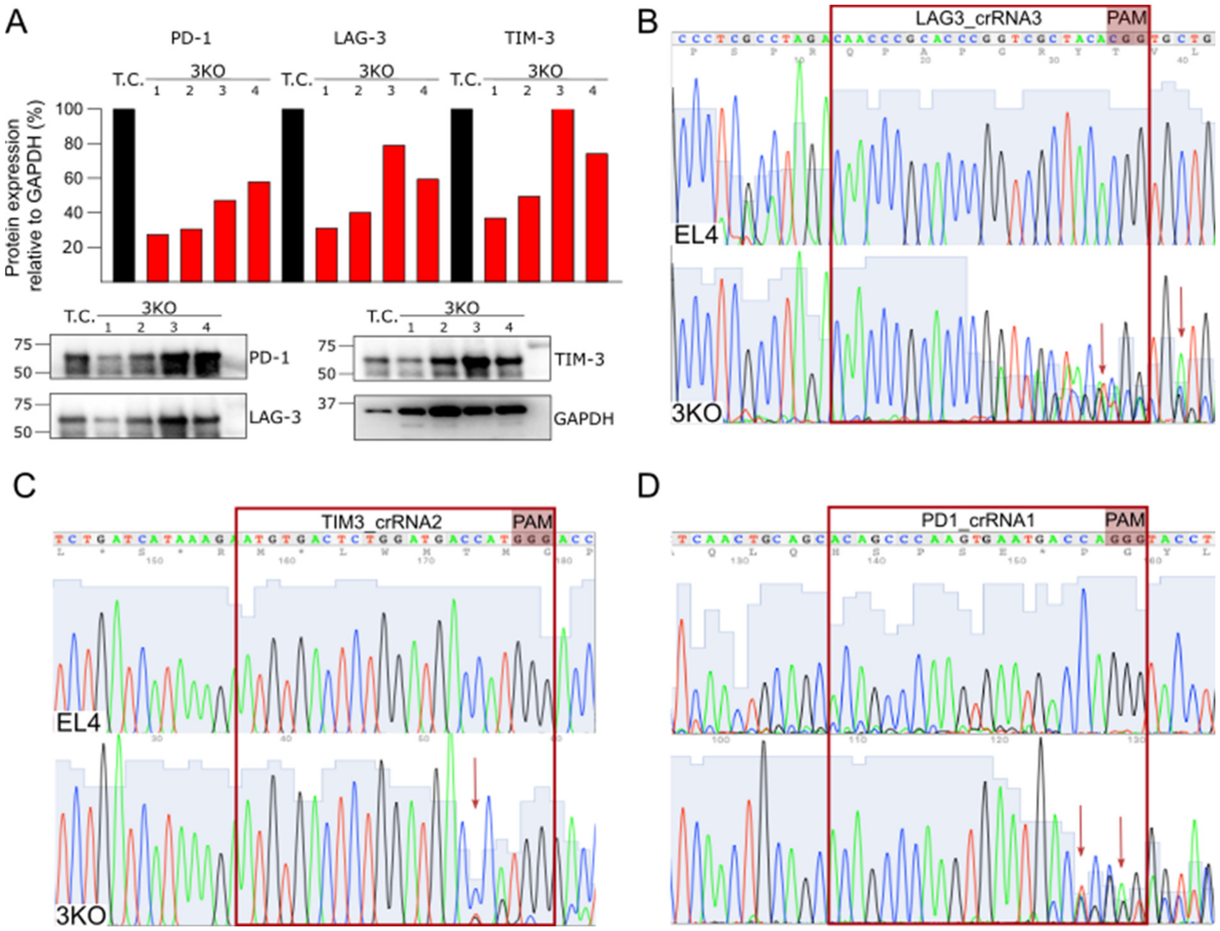

Supplementary Figure S1. CRISPR/Cas9 gene editing of LAG-3, TIM-3 and PD-1

(A) Western blot representing the expression of LAG-3, TIM-3, and PD-1 expression of transfection control EL4 (T.C.) and EL4 single cells clones transfected with all three crRNA (3KO, clones 1-4). Transfected EL4 cells were firstly transfected with CRISPR-Cas9 system and clones were derived after single cell cloning as describe in material and methods section. Clones were lysed in laemmli buffer and analyzed through western blot. PVDF membrane were incubated with anti PD-1, TIM-3, LAG-3 and GADPDH antibody and signal was revealed through HRP-conjugated secondary antibodies and ECL substrate. Luminescent signal was detected and measured. (B, C and D). DNA sequence of LAG-3, TIM-3, and PD-1, on the annealing region of the crRNA of wild type (up) and transfected (low) EL4 cells. Sequences were performed by using forward primers: PD1_fw: GTTATGCTGAAGGAAGAGCCCTGC; LAG3_fw: TCTCTCTCCCTTTGTCCGGC; TIM3_fw: CTATCTACACCTGGGGCACTTG.

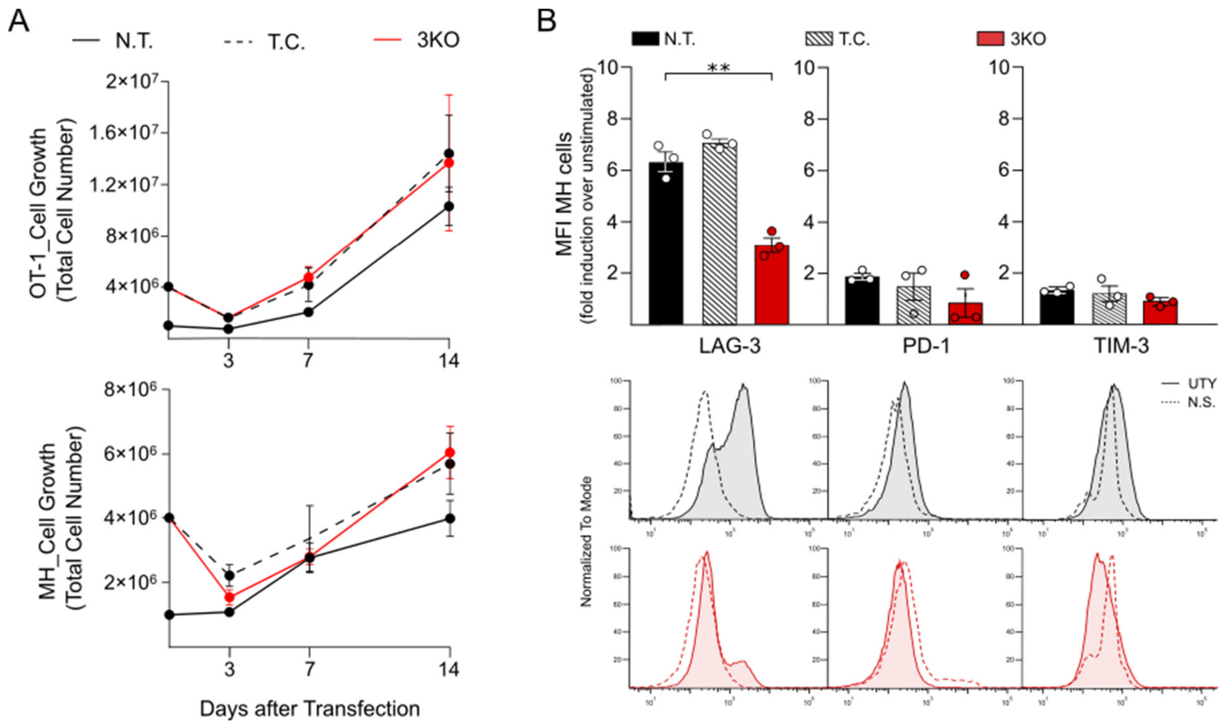

Supplementary Figure S2. Cell growth after transfection and TIRs expression after 72 hours CD8⁺ T cell stimulation

(A) Cell growth of OT-1 and MH $CD8^+$ T cells after CRISPR-Cas9 transfection. After transfection, cells were followed for 14 days and T cell expansion was measured. (B) Upper panel. Mean Fluorescence Intensity (MFI) of LAG-3, PD-1, and TIM-3 in MataHari (MH) $CD8^+$ T cells after 72h stimulation with UTY peptide. Values correspond to the fold induction of UTY stimulated MH $CD8^+$ T cells over the unstimulated. Lower panel. Representative dot plot curves for LAG-3, PD-1, and TIM-3 intensity. MH $CD8^+$ T cells were stained with LAG-3, PD-1, and TIM-3 specific fluorescent antibodies and analyzed by cytofluorimetry. Black line (straight and dotted) corresponds to N.T.; red line (straight and dotted) corresponds to 3KO. Bars are representative of three independent experiments with MH $CD8^+$ T cells derived from three different animals. N.T., not transfected; T.C., transfection control; 3KO, triple knock out for LAG-3, PD-1, and TIM-3 gene; N.S., not stimulated. Statistical analysis: one-way Anova with Post-hoc Test. **p<0,01.

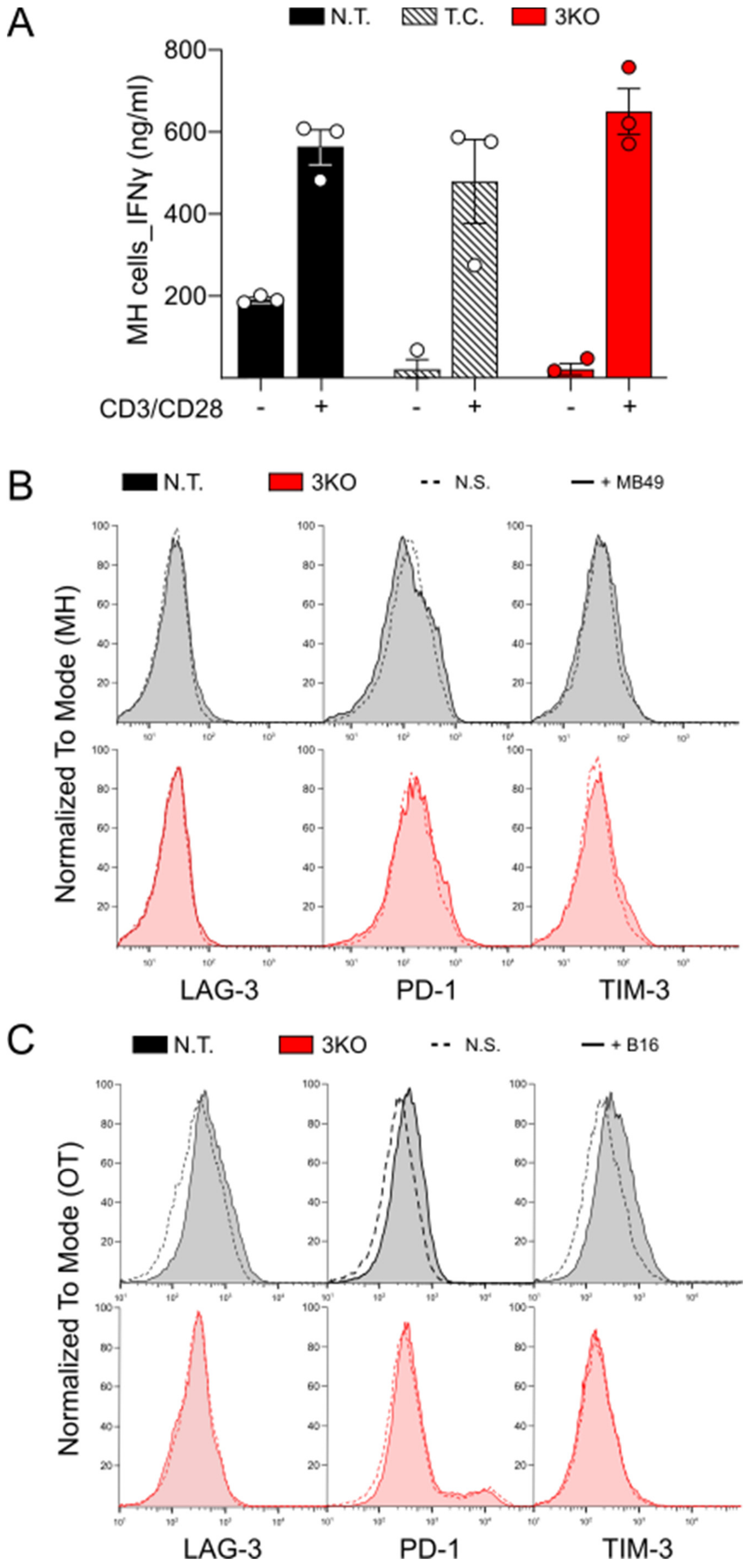

A
N.T.
T.C.
3KO
MH cells_IFNγ (ng/ml)
800
600
400
200
CD3/CD28
-
+
-
+
-
+
B
N.T.
3KO
N.S.
+ MB49
Normalized To Mode (MH)
LAG-3
PD-1
TIM-3
C
N.T.
3KO
N.S.
+ B16
Normalized To Mode (OT)
LAG-3
PD-1
TIM-3

Supplementary Figure S3. MB49 and B16 do not induce TIRs expression after 24 hours incubation with CD8⁺ T cells

(A) Interferon γ (IFNγ) production after stimulation with anti-CD3/CD8 antibodies. MH CD8⁺ T cells were stimulated for 72 hours and supernatant analyzed through ELISA for the IFNγ content. (B) Representative dot plot curves for LAG-3, PD-1, and TIM-3 intensity. 10^5 MH CD8⁺ T cells were incubated for 24 hours in presence of 10^4 MB49 tumor cells. After 24 hours MH CD8⁺ T cells were isolated and stained with LAG-3, PD-1, and TIM-3 fluorescent antibodies and analyzed by cytofluorimetry. Black line (straight and dotted) corresponds to N.T.; red line (straight and dotted) corresponds to 3KO. Dot plots are representative of at least three independent experiments with MH CD8⁺ T cells derived from three different animals. (C) Representative dot plot curves for LAG-3, PD-1, and TIM-3 intensity. 10^5 OT-1 CD8⁺ T cells were incubated for 24 hours in presence of 10^4 B16-OVA tumor cells. After 24 hours, MH CD8⁺ T cells were isolated and stained with LAG-3, PD-1, and TIM-3 fluorescent antibodies and analyzed by cytofluorimetry. Black line (straight and dotted) corresponds to N.T.; red line (straight and dotted) corresponds to 3KO. Dot plots are representative of at least three independent experiments with MH CD8+ T cells derived from three different animals.

N.T., not transfected; T.C., transfection control; 3KO, triple knock out for LAG-3, PD-1 and TIM-3 gene; N.S., not stimulated.

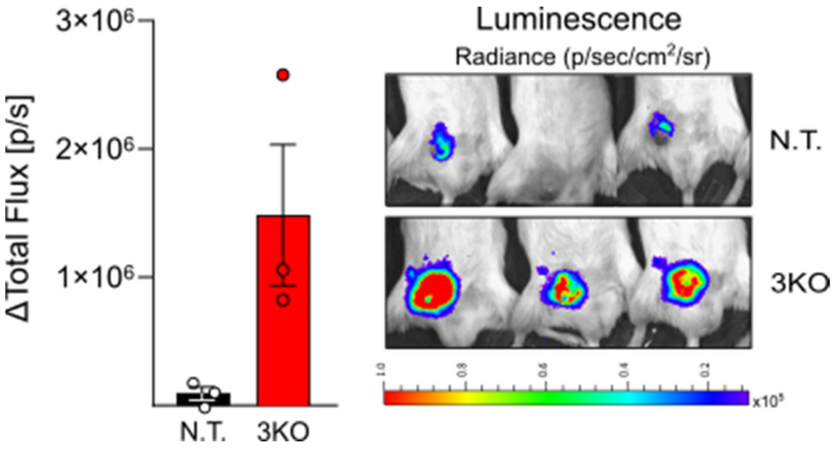

Supplementary Figure S4. Triple edited OT-1 T cells showed enhanced tumor infiltration

(A) Quantification of Renilla Luciferase (RLuc) signal at day 8 after injection of OT-1 T cells expressing constitutively RLuc. (B) BLI analysis of tumor infiltration at day 8 after ATT (n=3 per group). (BLI), Bioluminescent imaging; (N.T.), not transfected; (3KO), triple knock out for PD-1, LAG-3, and TIM-3 gene; (p/s), photons/second; (p/sec/cm²/sr), photons/second/cm²/ steradian.

Supplementary Materials and Methods

Western blot Analysis

EL4 cells transduced and not transduced were collected for western blot detection of PD-1, LAG-3, TIM-3 and GAPDH (housekeeping protein) levels. Protein samples were resolved by 10% SDS-PAGE and transferred on to PVDF (Immobilon™-P) membranes. The membranes

were blocked with 5% BSA in 1x TBS with 0.3% Tween 20 for 30 min at 42 °C. Then, the membranes were incubated overnight at 4 °C with primary antibodies (LAG3 (E5S8V) Rabbit mAb #80282, Cell Signaling, USA; PD-1 (D7D5W) XP® Rabbit mAb (Mouse Specific) #84651, Cell Signaling, USA; Mouse TIM-3 Antibody #MAB15291-SP, R&D system, USA; Anti-GAPDH antibody, Mouse monoclonal # G8795-100uL, Sigma-Aldrich, Merck KGaA, Germany) and 1 h with HRP-conjugated secondary antibody (Anti-Rabbit IgG (Fc specific) #AB3700853-2MG, Sigma-Aldrich, Merck KGaA, Germany; Rabbit Anti-Mouse IgG Antibody, HRP conjugate # AP160P, Sigma-Aldrich, Merck KGaA, Germany). The blots were visualized in ECL solution (Clarity Western ECL Substrate, #1705061, Bio-Rad Laboratories, USA) for 1 min and images were captured on Luminescent Image Analyser (ChemiDoc Imaging Systems, Bio-Rad Laboratories, USA).

BioLuminescent imaging (BLI)

In vivo BLI was performed using a Xenogen IVIS 200 (PerkinElmer, Waltham, MA, United States) after mice were anesthetized with isoflurane (Baxter, Deerfield, IL, United States) in an XGI-8 anesthesia system (PerkinElmer, Waltham, MA, United States). In order to analyze Renilla luciferase signals (T cell engraftment, migration and persistence), mice were imaged 3 minutes after intravenous injection of freshly prepared coelenterazine (Biosynth Ltd, Newbury, United Kingdom) dissolved in 100% DMSO (Sigma-Aldrich, Merck KGaA, Darmstadt, Germany) and diluted in PBS (#K813-500 mL, VWR) (100 µg/100 µL per mouse). Images were acquired for 5 minutes using small binning. All data were analyzed using Living Image analysis software (PerkinElmer, Waltham, MA, United States). The signal strength was quantified by photon/s/cm2/steradian after digital setting of equal regions of interest.
